# Upregulation of microRNA-125b-5p alleviates acute liver failure by regulating the Keap1/Nrf2/HO-1 pathway

**DOI:** 10.3389/fimmu.2022.988668

**Published:** 2022-10-04

**Authors:** Ya-Chao Tao, Yong-Hong Wang, Meng-Lan Wang, Wei Jiang, Dong-Bo Wu, En-Qiang Chen, Hong Tang

**Affiliations:** ^1^ Center of Infectious Diseases, West China Hospital, Sichuan University, Chengdu, China; ^2^ Division of Infectious Diseases, State Key Laboratory of Biotherapy, Sichuan University, Chengdu, China

**Keywords:** acute liver failure, acute-on-chronic liver failure, microRNA-125b-5p, Kelch-like ECH-associated protein 1, high-throughput sequencing, inflammation

## Abstract

**Background:**

Acute liver failure (ALF) and acute-on-chronic liver failure (ACLF) are the two most common subtypes of liver failure. They are both life-threatening clinical problems with high short-term mortality. Although liver transplantation is an effective therapeutic, its application is limited due to the shortage of donor organs. Given that both ACLF and ALF are driven by excessive inflammation in the initial stage, molecules targeting inflammation may benefit the two conditions. MicroRNAs (miRNAs) are a group of small endogenous noncoding interfering RNA molecules. Regulation of miRNAs related to inflammation may serve as promising interventions for the treatment of liver failure.

**Aims:**

To explore the role and mechanism of miR-125b-5p in the development of liver failure.

**Methods:**

Six human liver tissues were categorized into HBV-non-ACLF and HBV-ACLF groups. Differentially expressed miRNAs (DE-miRNAs) were screened and identified through high-throughput sequencing analysis. Among these DE-miRNAs, miR-125b-5p was selected for further study of its role and mechanism in lipopolysaccharide (LPS)/D-galactosamine (D-GalN) -challenged Huh7 cells and mice *in vitro* and *in vivo.*

**Results:**

A total of 75 DE-miRNAs were obtained. Of these DE-miRNAs, miR-125b-5p was the focus of further investigation based on our previous findings and preliminary results. We preliminarily observed that the levels of miR-125b-5p were lower in the HBV-ACLF group than in the HBV-non-ACLF group. Meanwhile, LPS/D-GalN-challenged mice and Huh7 cells both showed decreased miR-125b-5p levels when compared to their untreated control group, suggesting that miR-125b-5p may have a protective role against liver injury, regardless of ACLF or ALF. Subsequent results revealed that miR-125b-5p not only inhibited Huh7 cell apoptosis *in vitro* but also relieved mouse ALF *in vivo* with evidence of improved liver histology, decreased alanine aminotransferase (ALT) and aspartate aminotransferase (AST) levels, and reduced tumor necrosis factor-α (TNF-α) and IL-1β levels. Based on the results of a biological prediction website, microRNA.org, Kelch-like ECH-associated protein 1 (Keap1) was predicted to be one of the target genes of miR-125b-5p, which was verified by a dual-luciferase reporter gene assay. Western blot results *in vitro* and *in vivo* showed that miR-125b-5p could decrease the expression of Keap1 and cleaved caspase-3 while upregulating the expression of nuclear factor (erythroid-derived 2)-like 2 (Nrf2) and heme oxygenase-1(HO-1).

**Conclusion:**

Upregulation of miR-125b-5p can alleviate acute liver failure by regulating the Keap1/Nrf2/HO-1 pathway, and regulation of miR-125b-5p may serve as an alternative intervention for liver failure.

## Introduction

Liver failure is a life-threatening clinical problem with high short-term mortality. It can present as acute liver failure (ALF, without pre-existing chronic liver disease), acute-on-chronic liver failure (ACLF, an acute deterioration of underlying chronic liver disease) or an acute decompensation of an end-stage liver disease (1). Liver transplantation is an effective therapeutic option, irrespective of the etiology of liver failure. However, the application of liver transplantation is limited due to the shortage of donor organs (2). Thus, liver failure is still a severe clinical challenge, and other interventions assisting alleviating liver injury are being explored.

ALF and ACLF are the most widely discussed owing to their high incidence worldwide. Better knowledge of the pathophysiology of these diseases can provide insights into novel therapies. It has been reported that the development of both ALF and ACLF is driven by immune dysfunction and inflammatory imbalance, although the conditions are distinct clinical entities (3–5). The immune and inflammatory status of the diseases is dynamic, progressing from intensive inflammation to the development of immunoparalysis (3, 4, 6). In the initial phase of ALF, immune cells participating in the innate response are activated to produce proinflammatory mediators, which can stimulate a systemic inflammatory response. Patients with ACLF display an excessive innate immune response, which is characterized by leukocytosis, neutrophilia and lymphopenia, together with high levels of inflammatory mediators (7, 8). Initial systemic inflammatory response syndrome (SIRS) due to acute insult and/or subsequent secondary infection due to immunoparalysis can lead to extrahepatic organ failure (4, 9, 10). Thus, strategies modulating immune and proinflammatory mediators can be potential targets to alleviate liver failure.

MicroRNAs (miRNAs) are a class of small endogenous noncoding interfering RNA molecules and can induce gene silencing and translational repression by binding specific sequences in target mRNAs, thereby playing key roles in biological processes and in the development of various diseases (11). Accumulating studies have demonstrated that miRNAs are involved in the modulation of immunity and inflammation, and regulation of these miRNAs may be potential therapeutics for clinical problems (12–14). For instance, inhibition of miR-34b-5p could attenuate inflammation and apoptosis in acute lung injury, and thus miR‐34b‐5p and its target progranulin might be a potential intervention pathway for the treatment of acute lung injury (15). Thus, the regulation of miRNAs and their target genes may improve the outcome of various diseases by regulating immunity and inflammation.

In the present study, we identified miRNAs that were differentially expressed in human HBV-ACLF tissues compared to HBV-non-ACLF tissues. Then, we further explored the role of a certain miRNA in the development of liver failure, aiming to determine whether the miRNA may improve liver failure by regulating intensive inflammation, hoping to pave the way for miRNA-targeting therapies for liver failure.

## Materials and methods

### Study population

Six patients with chronic HBV infection were enrolled. They all received antiviral therapy with nucleos(t)ide analogs. They were divided into HBV-non-ACLF (n=3) and HBV-ACLF (n=3) groups based on their liver histopathology and liver function. The histological assessments were performed using the METAVIR scoring system. Briefly, the degree of inflammation in the liver biopsies was assessed with the standard METAVIR histology activity index scoring system, defined as A0, no inflammation; A1, mild inflammation; A2, moderate inflammation, and A3, severe inflammation. The histological appearance of fibrosis was classified as F0 to F4, ranging from no fibrosis to cirrhosis (16).

HBV-non-ACLF referred to patients with chronic hepatitis B (CHB) who had abnormal liver function and mild-to-moderate inflammatory activity (≤A2) in the liver tissue due to HBV infection, but did not meet the diagnostic criteria of ACLF.

ACLF is diagnosed according to the recommendations of the Asian Pacific Association for the Study of the Liver (APASL) (1). Herein, patients who were previously diagnosed with CHB or cirrhosis belonged to the HBV-ACLF group if they manifested with jaundice (bilirubin>5 mg/dL), coagulopathy [prolonged international normalized ratio (INR)>1.5], encephalopathy, and ascites within 4 weeks. The histopathological findings of the HBV-ACLF group showed evident infiltration of inflammatory cells (A3), accompanied by the formation of pseudolobuli (F4). All HBV-ACLF patients later received liver transplantation.

Patients were excluded if they had any of the following conditions: (1) infections with other hepatitis viruses (including A, C, D, and E) or human immunodeficiency virus (HIV); (2) evidence of drug-induced liver injury, alcoholic liver disease, autoimmune liver diseases, severe systemic illnesses; (3) malignancies, such as hepatocellular carcinoma.

This study was carried out in accordance with the Declaration of Helsinki. All patients provided verbal informed consent. Detailed information about the study cohort is described in **Table 1**.

**Table 1:** Clinical characteristics of the two groups of patients.

Groups	Patients	Gender	Age(year)	ALT(IU/L)	AST(IU/L)	TBil(μmoL/mL)	INR	HBV-DNA (log10 IU/mL)	Inflammationactivity index	Fibrosis score
**HBV-non-ACLF**	Patient 1	male	39	50	44	9.9	1.24	4.049	A1	F0
	Patient 2	male	40	59	56	14.9	1.10	3.328	A2	F1
	Patient 3	male	46	114	93	14.0	1.05	3.755	A2	F1
**HBV- ACLF**	Patient 4	male	47	559	353	326.6	2.07	2.538	A3	F4
	Patient 5	male	52	442	293	396.3	1.95	2.661	A3	F4
	Patient 6	male	46	417	356	402.6	2.23	3.326	A3	F4

Upper limit of normal (ULN) of ALT: 50 IU/mL for male; ULN of TB: 28 μmol/L. HBV DNA<100 IU/mL is defined as undetectable serum HBV DNA.

ALT, Alanine aminotransferase; TBil, total bilirubin. INR, International Normalized Ratio.

### High-throughput sequencing

Total RNA was extracted using TRIzol reagent (Invitrogen, Carlsbad, CA, USA) according to the manufacturer’s instructions. The quantity and purity of total RNA were analyzed with an Agilent 2100 Bioanalyzer (Agilent, USA) with RIN> 6.5. Small RNAs of different length were separated using denaturing polyacrylamide gel electrophoresis (PAGE). Fragments between 18 and 30 nt in length were gel-purified and ligated to adaptors at both the 3′- and 5′-ends. The ligation products were subsequently reverse- transcribed into cDNA, and PCR amplification was performed using an Illumina sequencing kit (Illumina, USA) to generate a cDNA library according to previous studies (17, 18).

High-throughput sequencing was performed by Chengdu Life Baseline Technology Co., Ltd. As shown in **Supplement 1**, the raw read sequences were filtered to remove low-quality reads, 5′ adaptor contaminant reads, reads without 3’ adaptor sequences, reads containing poly (A) and adapter sequences, sequences <18 nt and sequences >32 nt. The clean reads were obtained and their length distributions were calculated using Fastx-Toolkit (19). Then, the clean reads were mapped and aligned to the human reference genome group and other small RNA databases using Bowtie2 software (20). The known miRNAs and novel miRNAs were identified and predicted using miRbase and miRDeep2, respectively (21, 22).

The miRNA expression levels between the two groups were compared to identify differentially expressed (DE)-miRNAs. The expression of miRNA was normalized using transcripts per million (TPM) as the following formula: TPM = (mapped read count/total clean read count) ×10^6^ (23). DE-miRNAs were defined as |log2 (fold change)| >1 between two groups with a false discovery rate (FDR) of <0.05.

### Bioinformatics analysis for DE-miRNAs

To understand the functions of these DE-miRNAs, their potential target genes were predicted by RNAhybrid (https://bibiserv.cebitec.uni-bielefeld.de/rnahybrid/) and miRanda (http://www.microrna.org/microrna/home.do) (24, 25). Only the target genes predicted by both methods were considered reliable targets for further analysis. Gene ontology (GO) functional enrichment analysis and Kyoto Encyclopedia of Genes and Genomes (KEGG) pathway enrichment analysis were performed on these target genes (26, 27). GO analysis was conducted to provide functional annotation for predicted target genes of miRNAs by analyzing the classifications of Biological Process, Cellular Component and Molecular Function. Pathway analysis was based on KEGG, which is a database resource for understanding the high-level functions and utilities of biological systems. *p*<0.05 was regarded as the cutoff to select significantly enriched terms.

### Cell culture and transfection

The human HCC cell line Huh7 was preserved in our laboratory. Cells were cultured in Dulbecco’s modified Eagle’s medium (DMEM, D6429, Sigma, USA) containing 10% fetal bovine serum (12103C, Sigma, USA) and 1% penicillin/streptomycin under standard culture conditions (a humidified 5% carbon dioxide incubator at 37°C).

The miR-125b-5p overexpression vector and negative control (NC) vector were designed and provided by *Heyuan Biotechnology* (OBIO, Shanghai, China). After growth to 60%-70% confluence in six-well plates, Huh7 cells were transfected with miR-125b-5p vector or NC vector using Lipofectamine™ 2000 (11668019, Invitrogen, USA). On the following day, the cells were incubated with normal medium.

### Flow cytometric analysis

Apoptotic cells were assessed using an Annexin V- Fluorescein isothiocyanate (FITC) apoptosis detection kit (APOAF, Sigma, USA) according to the manufacturer’s protocol. Huh7 cells were collected and washed twice with PBS. Then, the cells were resuspended in 500 μL of 1× binding buffer and stained with 5 μL of Annexin V-FITC conjugate and 10 μL of PI solution. After incubation for 15 min in the dark at room temperature, stained cells were analyzed by flow cytometry (BD Accuri™ C6 flow cytometer).

### Real−time quantitative PCR analysis

Total RNA was extracted from Huh7 cells and liver tissues using TRIzol Reagent (15596026, Invitrogen, USA) according to the manufacturer’s instructions. Reverse transcription was performed using a First Strand cDNA Synthesis Kit (B300537, Sangon Biotech, China). The expression of miR-125b-5p was determined by quantitative real-time PCR using a miRNA qPCR detection kit (B532461, Sangon Biotech, China), as previously reported (28). The forward primer sequence for miR-125b-5p was CGTCCCTGAGA- CCCTAACTTGTGA. The reverse primers for miR-125b-5p were universal adaptor primers designed and provided by Sangon Biotech Company (Shanghai, China). The level of miR-125b-5p was calculated using the relative quantification 2^-ΔΔCT^ method and normalized to the U6 transcript (Bio-Rad CFX Manager software), as previously described (29, 30)

### Animals

Male C57BL/6J mice (6-8 weeks old, weighing 20-25 g) were purchased from Huaxi Laboratory Animal Center of Sichuan University (Chengdu, China). All mice were maintained under controlled conditions (24°C, 55% humidity and 12-h day/night rhythm) and given free access to water and food. The mice received humane care under guidance from the Institutional Review Board in accordance with the Animal Protection Art of Sichuan University. After 1 week of acclimation, the mice were prepared for further study.

### Mouse model of acute liver failure

The mouse model of ALF was established using lipopolysaccharide (LPS) and D-galactosamine (D-GalN) as previously described (31). In brief, C57BL/6J mice were given 700 mg/kg D-GalN (G0500, Sigma, USA) and 10 μg/kg LPS (*Escherichia coli*, 0111:B4, L2630, Sigma, USA) by intraperitoneal injection. Mice in the present study were randomly divided into four groups (n = 8/group): a normal control group, an LPS/D-GalN group, an LPS/D-GalN+ negative control (NC) group and an LPS/D-GalN+ miR-125b-5p group.

The miR-125b-5p overexpression vector or negative control vector was administered to mice *via* tail vein prior to the establishment of ALF as previously reported (32, 33). Seven hours after ALF model establishment, all mice were sacrificed, and serum and liver samples were harvested and stored for further analysis.

### H&E staining

Liver tissue was obtained and fixed with 4% paraformaldehyde at 4°C for 48 h and then embedded in paraffin. After immobilization, samples were cut into sections and then stained with hematoxylin-eosin (HE) using a standard protocol. The sections were visualized under a light microscope, and representative images are presented.

### Biochemical detection of aminotransferase

Serum samples were collected from mice to detect alanine aminotransferase (ALT) and aspartate aminotransferase (AST) by an automatic biochemical analyzer.

### Enzyme-linked immunosorbent assay (ELISA)

The levels of serum tumor necrosis factor -α (TNF-α, EMC102a, NeoBioscience, China) and IL-1β (EMC001b, NeoBioscience, China) were detected using ELISA kits according to the manufacturer’s instructions.

### Western blotting

Protein was extracted from Huh7 cells and liver tissues. The isolated proteins were separated by polyacrylamide gel electrophoresis and then transferred onto polyvinylidene fluoride (PVDF) membranes. Subsequently, the blots were exposed to primary antibodies against Kelch-like ECH-associated protein 1 (Keap1, #8047, CST, USA), nuclear factor (erythroid-derived 2)-like 2 (Nrf2, sc-365949, Santa Cruz, USA), heme oxygenase-1 (HO-1, #43966, CST, USA), and cleaved caspase-3 (#9661, CST, USA). Anti-GAPDH (TA-08), anti-β-tubulin (TA-10) and anti-β-actin (TA-09) were used as internal references, and were purchased from Beijing Zhong Shan-Golden Bridge Biological Technology Co., Ltd. The images of the gels were captured in a Bio-Rad Image Lab (ChemiDoc™ MP Imaging System, Bio-Rad, California, USA).

### Dual-luciferase reporter gene assay

A biological prediction website, microRNA.org, was used to analyze the target genes of miR-125b-5p, and a dual-luciferase reporter gene assay was performed to detect whether miR-125b-5p extensively targeted Keap1. The full length of the 3′-untranslated region (3′UTR) (forward primer: GAGGAGTTGTGTTTGTGGAC; reverse primer: TGTAAAACGACGGCCAGT) of the Keap1 gene was amplified clonally. The wild-type (WT) vector of Keap1 (Keap1-WT) and a mutant type vector (Keap1-MUT) were constructed. Bioinformatics software was used to predict the binding sites between miR-125b-5p and Keap1. The vectors were cotransfected with miR-125b-5p mimic and mimic negative control into 293T cells according to the following groups: Keap1-WT+ miR-125b-5p mimic negative control (miR-NC), Keap1-WT+ miR-125b-5p mimic, Keap1-MUT+ miR-NC, and Keap1-MUT+ miR-125b-5p mimic. Firefly and Renilla luciferase activities were measured using a dual luciferase reporter gene detection kit. The experiment was repeated three times in each group.

### Statistical analysis

Statistical analysis was performed using SPSS 20.0 software. Data are presented as the mean± deviation (SD) determined from a minimum of three independent experiments each performed with triplicate cultures. One-way ANOVA was performed to evaluate the level of significance, and the results were considered statistically significant if *p*<0.05.

## Results

### Sequence analysis of small RNAs and identification of DE-miRNAs

**Figure 1:**
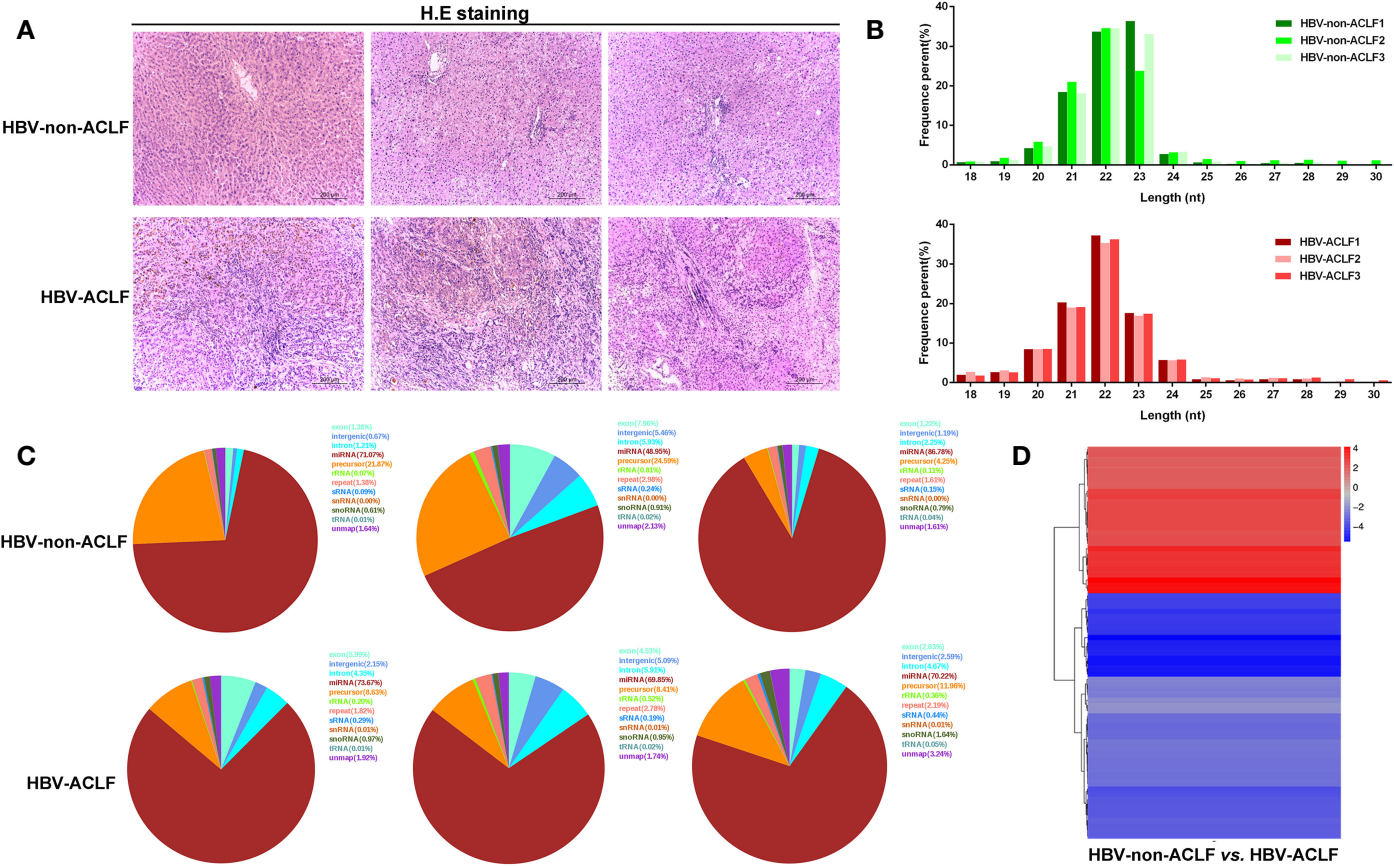
Results of high-throughput sequencing and identification of DE-miRNAs. **(A)** H&E staining of liver tissues from HBV-non-ACLF and HBV-ACLF groups; **(B)** Length distribution of the small RNA library; **(C)** Distribution of small RNAs among different categories; **(D)** Heatmap of DE-miRNAs constructed by comparing the levels of miRNAs between the two groups.

Six liver tissues were obtained and categorized into HBV-non-ACLF and HBV-ACLF groups based on the severity of liver injury (**Table 1**, **Figure 1A**). The sequences of small RNAs ranged from 18-30 nt in length, of which the majority were 21-23 nt long, and the 22-nt small RNAs were the most abundant (**Figure 1B**). After removal of the adaptor, insert, poly (A) tail and short RNAs of <18 nt, a total of 59,160,359 and 49,723,211 clean reads were obtained for the two groups, respectively (**Supplement 2**). Small RNA sequences were searched against the GenBank, Rfam, and Repbase databases by Bowtie2 software, rRNA, snRNA, snoRNA, tRNA, sRNA, etc., were annotated and removed, and the unannotated RNAs were subjected to further analyses for miRNA identification (**Figure 1C**). We used the software miRbase and miRDeep2 to map the retained sequence reads to identify candidate miRNAs.

DE-miRNAs were obtained when they met the criteria of |log2 (fold change)|>1 between the two groups and FDR<0.05, and these DE-miRNAs are presented in a heatmap (**Figure 1D**). Compared with the HBV-non-ACLF group, the levels of 28 miRNAs were increased, while the levels of 47 miRNAs were decreased in the HBV-ACLF group. Some of the upregulated and downregulated miRNAs are shown in **Tables 2** and **3**, respectively.

**Table 2:** Ten of the upregulated DE-miRNAs in the HBV-ACLF group compared to the HBV-non-ACLF group.

Gene ID	HBV-non-ACLF vs. HBV-ACLF		Regulation
	Log2(HBV-ACLF/HBV-non-ACLF)	FDR
hsa-miR-1827	3.779564	0.020201	Up
hsa-miR-3934-5p	3.743929	0.020344	Up
novel_mir70	2.968886	0.020482	Up
hsa-miR-548ba	2.846975	0.023141	Up
hsa-miR-329-3p	2.812766	0.005075	Up
hsa-miR-3690	2.577176	0.002611	Up
hsa-miR-147b	2.303872	0.012293	Up
hsa-miR-6718-5p	2.292068	0.000115	Up
hsa-miR-143-5p	2.289669	0.001049	Up
hsa-miR-155-3p	2.301391	0.011031	Up

FDR, false discovery rate.

**Table 3:** Twenty of the downregulated DE-miRNAs in the HBV-ACLF group compared to the HBV-non-ACLF group.

**Gene ID**	**HBV-non-ACLF vs. HBV-ACLF**		**Regulation**
	**Log2(HBV-ACLF/HBV-non-ACLF)**	**FDR**
hsa-miR-378i	-5.58567	1.40E-06	Down
novel_mir3	-5.16057	2.09E-07	Down
hsa-miR-6130	-5.13246	3.08E-06	Down
novel_mir143	-4.98430	6.36E-17	Down
hsa-miR-216a-5p	-4.80066	0.00012	Down
novel_mir211	-4.73787	7.88E-05	Down
hsa-miR-216b-5p	-4.73061	8.89E-07	Down
hsa-miR-216b-3p	-4.3374	1.11E-05	Down
hsa-miR-483-3p	-4.17394	1.89E-09	Down
hsa-miR-4686	-4.08130	1.43E-07	Down
hsa-miR-3591-3p	-3.93922	3.67E-05	Down
hsa-miR-3591-5p	-3.93449	1.23E-08	Down
hsa-miR-375	-3.88737	1.75E-08	Down
hsa-miR-505-5p	-3.56423	1.15E-05	Down
hsa-miR-122-5p	-3.33444	0.002364	Down
hsa-miR-483-5p	-3.30266	1.65E-05	Down
hsa-miR-338-5p	-3.20726	0.00599	Down
hsa-miR-1295a	-3.12447	1.40E-06	Down
hsa-miR-192-3p	-2.85876	2.09E-05	Down
hsa-miR-125b-5p	-2.58513	0.000155	Down

FDR, false discovery rate.

### GO and KEGG enrichment analysis

To further understand the roles of miRNAs in the progression of liver injury, GO and KEGG analyses of putative target genes of these DE-miRNAs were performed. GO describes genes from three aspects, namely molecular function (MF), cellular component (CC) and biological process (BP).

**Figure 2:**
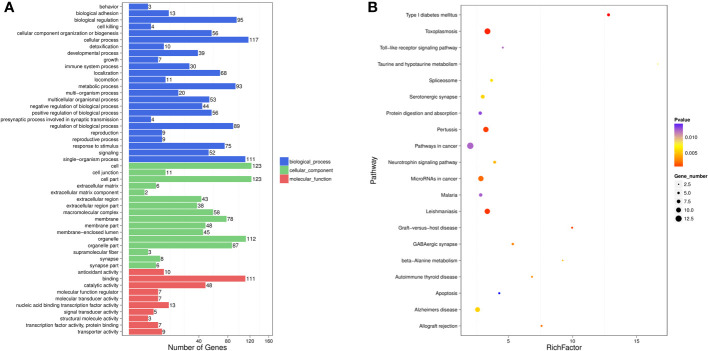
GO functional enrichment analysis **(A)** and KEGG pathway analysis **(B)** for DE-miRNAs obtained from the comparsion between HBV-non-ACLF and HBV-ACLF groups. Rich actor is the ratio of the DE gene number to the total gene number in a certain pathway. The size and color of the dots represent the gene number and range of the p value, respectively.

Among the DE-miRNA target genes, “binding” was the most represented in the MF category, followed by “catalytic activity”, “nucleic acid binding transcription factor activity”, “antioxidant activity” and “transporter activity”. For the CC category, target genes mostly participated in the “cell”, “cell part”, “organelle”, “organelle part” and “membrane”. Genes involved in the “cellular process”, “single-organism process”, “biological process”, “metabolic process” and “regulation of biological process” were notably represented in the BP category (**Figure 2A**). In the KEGG analysis, target genes were primarily enriched in the “pathways in cancer”, “toxoplasmosis”, “pertussis”, “leishmaniasis” and “microRNAs in cancer” (**Figure 2B**).

### Protective action of miR-125b-5p against ALF both *in vitro* and *in vivo*

We previously proved that serum miR-125b-5p was associated with the severity of HBV-related liver damage, and high serum miR-125b-5p might serve as a predictor for poor outcomes in HBV-ACLF cases (28). In the present study, we observed that the levels of hepatic miR-125b-5p were decreased with the aggravation of HBV-induced liver injury based on miRNA array data. Meanwhile, we verified the expression of miR-125b-5p using 20 pairs of liver tissues from patients with HBV-non-ACLF and HBV-ACLF and confirmed that the levels of hepatic miR-125b-5p were lower in HBV-ACLF patients than in HBV-non-ACLF patients (*p*<0.001, **Supplement 3A**). In addition, the levels of miR-125b-5p were decreased in LPS/D-GalN-challenged Huh7 cells (*p*<0.01, **Supplement 3B**) and mice (*p*<0.001, **Supplement 3C**) compared to those in untreated control groups. Thus, it was well-founded to speculate that miR-125b-5p might play an important role in the development of liver failure, regardless of ACLF or ALF, and we focused on this miRNA for further study.

**Figure 3:**
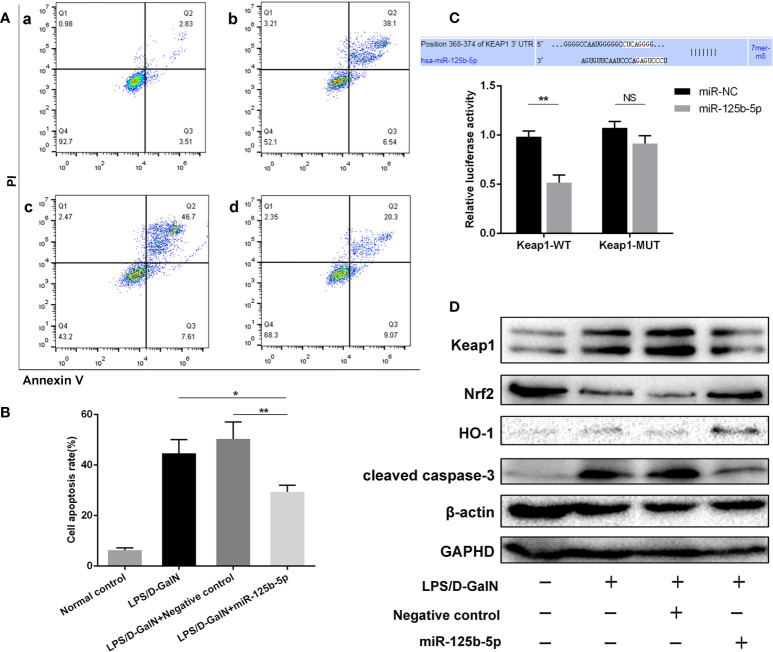
Role and mechanism of miR-125b-5p in injured Huh7 cells *in vitro*. **(A)** The cell apoptosis rate was calculated by flow cytometry. a: Normal control; b: LPS/D-GalN; c: LPS/D-GalN+ Negative control vector; d: LPS/D-GalN+ miR-125b-5p. **(B)** The cell apoptosis rate was calculated and compared. **(C)** A luciferase reporter assay was performed to identify the binding sites between human Keap1 and hsa-miR-125b-5p. **(D)** The expression levels of Keap1, Nrf2, HO-1 and cleaved caspase-3 were evaluated by western blotting. NS, no significance. *p< 0.05, **p < 0.01.

To determine the role of miR-125b-5p, Huh7 cells were transfected with miR-125b-5p overexpression vector or negative control vector. The expression of miR-125b-5p was significantly increased in the cells of the experimental group compared with the control group (*p*<0.001, **Supplement 3D**). Twenty-four hours after transfection with the vector, Huh7 cells were treated with LPS (20 ng/mL)/D-GalN (3 mol/mL) for 36 hours. Cell apoptosis was analyzed using annexin-V flow cytometry. As shown in **Figures 3A, B**, the apoptosis rates were significantly lower in cells transfected with miR-125b-5p vector before LPS/D-GalN addition than in cells only treated with LPS/D-GalN (29.41% ± 2.61% vs. 44.63% ± 5.45%, *p*<0.05) and in cells transfected with negative control vector (29.41% ± 2.61% vs. 50.39% ± 6.71%, *p*<0.01), demonstrating that miR-125b-5p may help to alleviate acute liver injury *in vitro* by inhibiting cell apoptosis.

**Figure 4:**
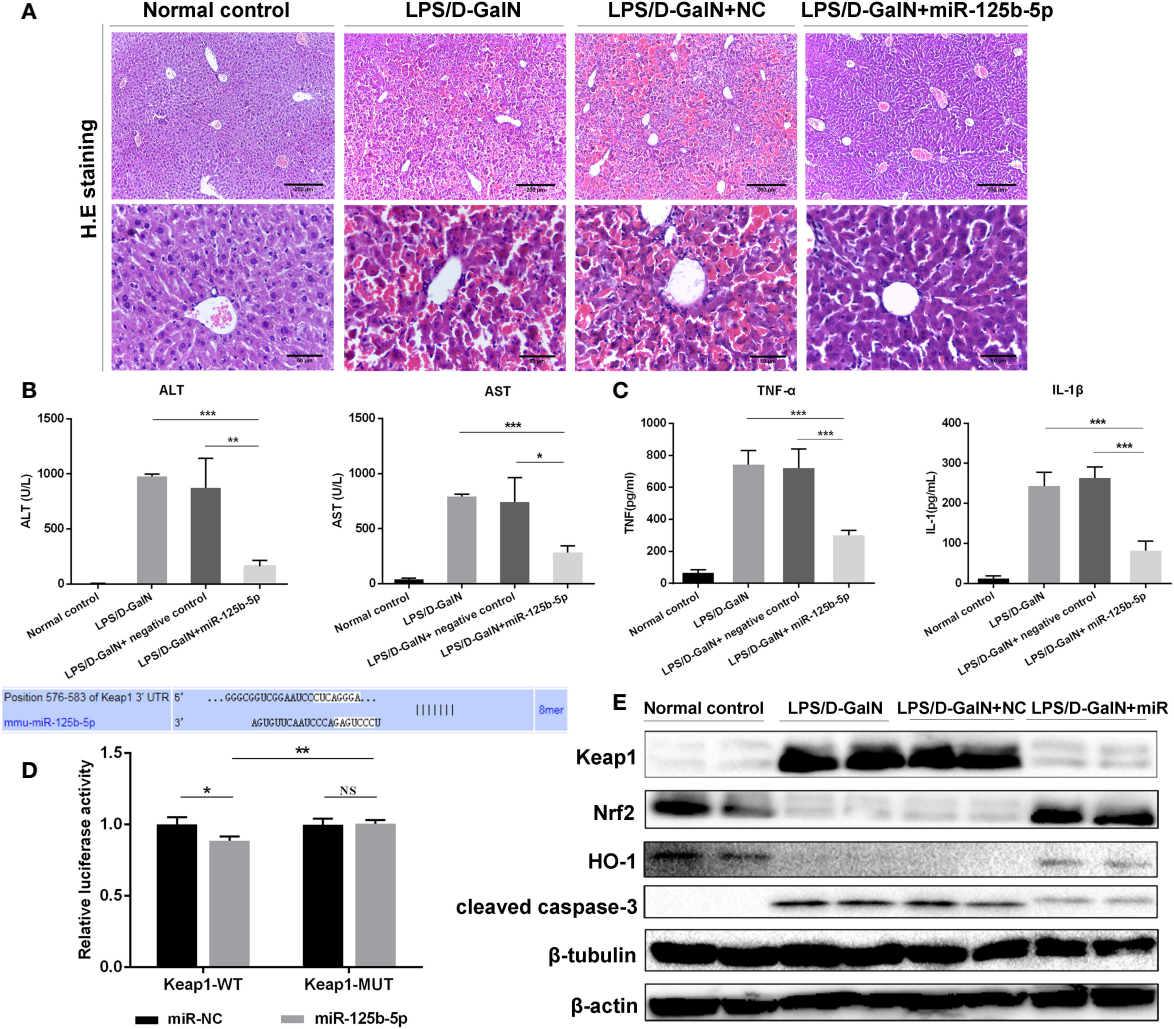
Role and mechanism of miR-125b-5p in LPS/D-GalN- induced ALF *in vivo*. **(A)** H&E staining of liver tissue. **(B)** Detection of serum ALF and AST. **(C)** Detection of two serum proinflammtory mediators, TNF-α and IL-1 β, using ELISA. **(D)** A luciferase reporter assay was performed to identify the binding sites between mouse Keap1 and mmu-miR-125b-5p. **(E)** The expression levels of Keap1, Nrf2, HO-1 and cleaved caspase-3 were evaluated by western blotting. NC, negative control. NS, no significance. *p<0.05, **p <0.01, ***p <0.001.

To further identify the protective role of miR-125b-5p in ALF *in vivo*, we established mouse ALF through intraperitoneal injection of LPS/D-GalN and assessed the improvements in liver histopathology and serum biochemical indicators after administration of the miR-125b-5p overexpression vector. The liver histopathological characteristics of the negative control groups showed no significant differences from those of the LPS/D-GalN-induced ALF groups, whereas administration of the miR-125b-5p vector improved the liver histopathology based on the retained liver structure, decreased inflammatory cell infiltration and reduced hemorrhage (**Figure 4A**). In addition, miR-125b-5p also reduced ALT and AST levels in mice treated with LPS/D-GalN (*p*<0.001, **Figure 4B**). Serum TNF-α and IL-1β levels were enhanced in the LPS/D-GalN-induced ALF group. In contrast, significant decreases in the two proinflammatory cytokines were observed in the miR-125b-5p treated group (*p*<0.001, **Figure 4C**).

### MiR-125b-5p alleviated ALF by regulating the Keap1/Nrf2 signaling pathway

A dual-luciferase reporter gene assay was performed to detect whether miR-125b-5p targeted Keap1 through the potential binding site predicted by TargetScan. The luciferase assay activity levels were reduced in the miRNA-125b-5p group compared with the miR-NC group, demonstrating that the 3’−UTR of human Keap1 mRNA was the binding site of miRNA-125b-5p, and unchanged levels of luciferase activity in the presence of the mutated 3’−UTR of Keap1 further confirmed that Keap1 is the target of miR-125b-5p (**Figure 3C**). We then investigated whether the Keap1 protein and its involved signaling pathway were regulated by miR-125b-5p *in vitro*. Overexpression of miRNA-125b-5p significantly suppressed the protein expression of Keap1. The Keap1/Nrf2 pathway plays a key role in mitigating acute liver injury (34). Our study found that the expression levels of Nrf2 and one of its downstream proteins, HO-1, were upregulated, whereas cleaved caspase-3 was decreased in miRNA-125b-5p- overexpressing cells compared to the control groups (**Figure 3D**).

We further explored whether miR-125b-5p could improve ALF *in vivo* by targeting Keap1 and its downstream proteins. The dual-luciferase reporter gene assay confirmed the direct binding of miR-125b-5p with the 3’-UTR of mouse Keap1 but not with the mutated 3’-UTR (**Figure 4D**). Subsequently, we found elevated levels of Nrf2 and HO-1 in miR-125b-5p-treated mice (**Figure 4E**). The expression of cleaved caspase-3 was significantly decreased in mice administered the miR-125b-5p overexpression vector. Thus, miR-125b-5p can regulate Keap1, which leads to the activation of Nrf2 signaling and hence alleviates LPS/D-GalN-induced ALF.

## Discussion

Liver failure is a challenging condition with high mortality; thus, the development of novel therapeutic agents for liver failure is crucial. Both ACLF and ALF are driven by dysfunctional immunity and excessive inflammation. Hence, strategies targeting immunity and inflammation may provide new insight into the treatment of liver failure.

In the present study, we screened DE-miRNAs in human liver tissues from patients with HBV-non-ACLF and HBV-ACLF using high-throughput sequencing. To the best of our knowledge, this is the first study that systematically quantifies the expression features of miRNAs by high-throughput sequencing analysis in human HBV-related liver tissue.

A total of 75 DE-miRNAs were ultimately obtained, and subsequent GO analysis revealed that most target genes of these DE-miRNAs were involved in “binding”, “cell part”, “cellular process” and related categories. KEGG analysis showed that target genes predominantly participated in the “pathways in cancer”, “toxoplasmosis”, and “microRNAs in cancer”. Although the functions of these DE-miRNAs in the development of liver failure remain unclear, several lines of evidence from previous findings suggest that our results are biologically reasonable. For instance, in the present study, the level of hepatic miR-122-5p was downregulated with the aggravation of liver injury. Previous study proved that circulating miR-122-5p might function as a new biomarker for CHB patients with normal or nearly normal ALT levels (35), and miR-122-5p knockdown protected against acetaminophen-induced liver injury (36). Likewise, miR-192-5p was downregulated when liver injury worsened in the present study, while a previous study proved that mR-192-5p was upregulated in serum during liver injury, and downregulation of miR-192-5p might protect against liver cell death (37). Overall, it seems that DE-miRNAs may participate in the development of liver injury, and some of them may be potential targets for the treatment of liver failure.

Of these DE-miRNAs, miR-125b-5p was the focus of further investigation based on our previous findings and preliminary results. We previously documented that circulating miR-125b-5p might be a novel biomarker for liver injury, and high serum miR-125b-5p levels might predict a poor outcome in HBV-ACLF patients (28). In the present study, we preliminarily observed that the hepatic miR-125b-5p levels were lower in the HBV-ACLF group than in the HBV-non-ACLF group. Meanwhile, LPS/D-GalN-challenged mice and Huh7 cells both showed decreased miR-125b-5p levels compared to their untreated control groups. Therefore, it is reasonable to speculate that miR-125b-5p may have a protective role against liver injury, regardless of the presence of ACLF or ALF.

Subsequent experiments were performed to determine the role of miR-125b-5p in acute liver injury both *in vitro* and *in vivo*. We found a reduced apoptosis rate of injured Huh7 cells after transfection with the miR-125b-5p vector, demonstrating that miR-125b-5p was able to ameliorate acute liver injury. Later, using a mouse ALF model induced by LPS/D-GalN, we observed severely damaged liver histology and elevated ALT/AST levels in ALF mice, whereas upregulation of miR-125b-5p reversed the impacts of liver failure, in line with a previous study in which miR-125b-5p was proven to protect against paracetamol- and FAS-induced toxicity in hepatocytes (38).

We next explored the underlying mechanism of miR-125b-5p in relieving ALF. Patients with liver failure, either ALF or ACLF, display evidence of a proinflammatory state in the initial stage. Targeting factors that are involved in the inflammatory response may be an alternative therapeutic for liver failure. Immune dysfunction in ALF and ACLF shares many features with sepsis (3, 4, 39). During sepsis, miRNAs are crucial regulators of immune cell function and participate in the inflammatory response by regulating the production of inflammatory factors, the vascular barrier and endothelial function (40–42). Thus, dysregulated miRNAs may also be involved in the inflammatory response during liver failure. It has been reported that miR-125b-5p may directly or indirectly participate in inflammation (43–45). MiRNA-125b-5p elevation can restrain the inflammatory response and protect against sepsis-induced acute lung injury (43). Correspondingly, dramatic decreases in the levels of serum TNF-α and IL-1β in mice treated with miR-125b-5p were observed in our study; thus, we speculated that miR-125b-5p may alleviate ALF by suppressing inflammation.

Keap1, as a putative target of miR-125b-5p, was verified using a luciferase activity assay in the present study. There was a negative correlation between miR-125b-5p and Keap1 expression in Huh7 cells and liver tissues; thus, miR-125b-5p targeted and negatively regulated Keap1. The Keap1/Nrf2 signaling pathway plays a pivotal role in the maintenance of intracellular redox homeostasis and the regulation of inflammation. Under homeostatic conditions, Nrf2 is mainly retained by Keap1 in the cytoplasm. Upon oxidative stress, Keap1 is inactivated, and Nrf2 dissociates from Keap1. Nrf2 then translocates to the nucleus and binds to antioxidant response elements (AREs), leading to the expression of diverse antioxidant and metabolic genes, such as glutathione S-transferase (GST) and heme oxygenase 1 (HO-1) (46). HO-1 is a critical protective enzyme that inhibits the release of proinflammatory cytokines and activates the anti-inflammatory cytokines (47). The Nrf2/HO-1 axis has been found to reduce the levels of TNF-α, IL-6 and IL-1-β and play a major role in anti-inflammatory function (48).

In the present study, we found that LPS/D-GalN decreased hepatic miR-125b-5p levels to target Keap1 and inhibit the Nrf2 pathway, triggering liver inflammation and cell apoptosis. In contrast, upregulation of miR-125b-5p decreased the level of Keap1 and promoted the expression of Nrf2 and its target HO-1. Hence, miR-125b-5p protects against LPS/D-GalN- induced ALF by targeting Keap1 and disrupting the interaction between Nrf2 and Keap1, followed by the activation of the Nrf2/HO-1 axis, thereby decreasing the levels of TNF-α and IL-1-β and inhibiting cell apoptosis, which was in accordance with previous evidence that regulation of the Keap1/Nrf2/HO-1 signaling pathway could alleviate acute liver injury (34, 49, 50).

However, only miR-125b-5p was investigated herein, and further studies are needed to disclose the molecular mechanisms by which other miRNAs, individually or cooperatively, contribute to the development of liver injury. Moreover, it may be better to establish a miRNA-network diagram targeting inflammation in the development of liver failure, which may provide new insight into the pathophysiology of liver failure and lay a basis for the study of new therapeutics.

In conclusion, our study provides evidence that miR-125b-5p can alleviate LPS/D-GalN-induced ALF by regulating the Keap1/Nrf2/HO-1 signaling pathway, and therefore, regulation of miR-125b-5p and its downstream target Keap1 may serve as an alternative intervention for liver failure.

## Data availability statement

The datasets presented in this study can be found in online repositories. The names of the repository/repositories and accession number(s) can be found below: https://www.ncbi.nlm.nih.gov/, PRJNA885574.

## Ethics statement

The animal study was reviewed and approved by animal ethics committee of Sichuan University.

## Author contributions

E-QC and HT proposed the study. Y-CT and Y-HW performed the research. Y-CT, M-LW, WJ, and D-BW contributed to the acquisition of data and drafting of the manuscript. All the authors contributed to the analysis and interpretation of data and approved the final version of the manuscript.

## Funding

This work was supported by the 1.3.5 project for disciplines of excellence, West China Hospital, Sichuan University (NO.ZYGD20009) and the Science and Technological Supports Project of Sichuan Province, China (NO. 2019YFS0028).

## Conflict of interest

The authors declare that the research was conducted in the absence of any commercial or financial relationships that could be construed as a potential conflict of interest.

## Publisher’s note

All claims expressed in this article are solely those of the authors and do not necessarily represent those of their affiliated organizations, or those of the publisher, the editors and the reviewers. Any product that may be evaluated in this article, or claim that may be made by its manufacturer, is not guaranteed or endorsed by the publisher.
