# Supplementary material for: Upregulation of microRNA-125b-5p alleviates acute liver failure by regulating the Keap1/Nrf2/HO-1 pathway
Source: Front Immunol. 2022 Oct 4;13:988668. doi: 10.3389/fimmu.2022.988668 (PMC9578503; doi:10.3389/fimmu.2022.988668)
Supplement: Supplementary file 1 [file DataSheet_1.docx]

**Supplement materials**

**Supplement 1.**


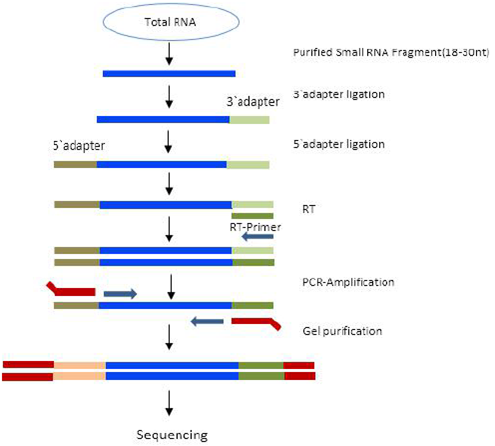


Supplement 1. General steps of high-throughput sequencing.

**Supplement 2.** Summary of sequencing data and quality filtering.

| **Groups** | **Samples** | **Clean Reads** | **Clean bases** | **Q20 (%)** | **GC (%)** |
| --- | --- | --- | --- | --- | --- |
|  | Patient 1 | 19,825,475 | 440,219,200 | 99.85% | 44.22% |
| **HBV-non-ACLF** | Patient 2 | 19,662,203 | 439,192,129 | 99.84% | 45.65% |
|  | Patient 3 | 19,672,681 | 436,911,646 | 99.83% | 44.37% |
|  | Patient 4 | 16,320,158 | 358,070,452 | 99.86% | 43.75% |
| **HBV-ACLF** | Patient 5 | 19,206,882 | 422,289,776 | 99.84% | 44.49% |
|  | Patient 6 | 14,196,171 | 314,384,396 | 99.67% | 43.99% |

**Supplement 3.**

**

**

Expression levels of miR-125b-5p in the liver and Huh7 cells were measured by quantitative real-time PCR. (A) Comparison of the hepatic miR-125b-5p levels between HBV-non-ACLF and HBV-ACLF patients; (B) Comparison of the miR-125b-5p levels between untreated Huh7 cells and LPS/D-GalN-challenged Huh7 cells; (C) Comparison of the hepatic miR-125b-5p levels between untreated normal mice and ALF mice; (D) Levels of miR-125b-5p in Huh7 cells transfected with miR-125b-5p overexpression vector. ***p* <0.01, ****p* <0.001.
